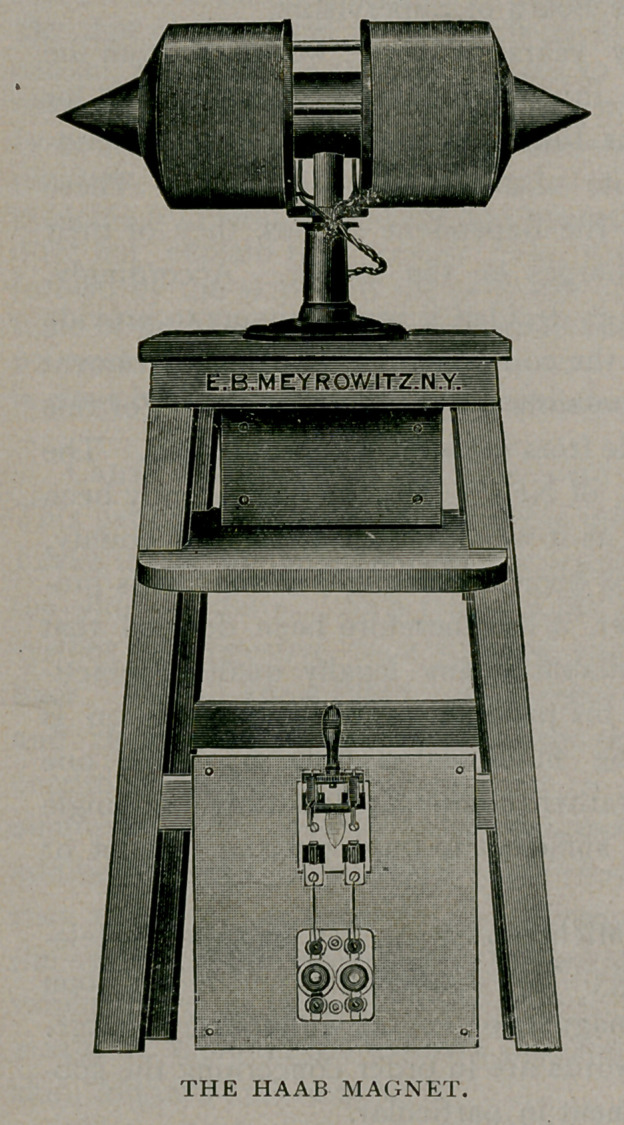# On a Form of the Haab Magnet

**Published:** 1900-05

**Authors:** Lucien Howe

**Affiliations:** Buffalo, N. Y.


### ON A FORM OF THE HAAB MAGNET.

By LUCIEN HOWE, M. D., Buffalo, N. Y.

THE main object of this brief note is to acquaint physicians of Buffalo and of Erie County with the fact that a powerful magnet for removing particles of iron from the eye has been placed at the disposal of the profession by the Board of Supervisors.

At intervals during twenty years or more, workmen from the shops and others who had been injured in this way have applied for relief at the Buffalo Eye and Ear Infirmary and partial or total blindness has often resulted in spite of every effort for relief. These unfortunates naturally swelled the dependent classes, they or their families with them becoming charges on the county. Accordingly, last year the Board of Supervisors decided it was economy to provide at least some one institution in the county with a suitable instrument for giving whatever relief was possible in the early stage. For this purpose, about $250 was set aside from the annual appropriation. The magnet was built by Meyrowitz, of New York, and has recently been placed in position. Moreover, as it was purchased by the county, and might be needed suddenly by poor or rich alike, as one needs protection from any sudden danger, it has therefore been decided that the magnet should be made available by any legally qualified practitioner of the county for any of his patients. The sole condition is that the instrument should be manipulated only by some one thoroughly familiar with its rather complicated setting, a single wrong turn of a switch being sufficient to burn out the magnet or injure the patient.

The Infirmary, at No. 673 Michigan Street, can be called by telephone night or day—Seneca 1253—and a competent person will soon be ready to manipulate the magnet, of course without any charge. With this explanation a few words are in order concerning the subject in general and this instrument in particular.

It was an old idea that particles of iron could be removed from the eye by means of a magnet. The popular notion still is that when such a fragment is imbedded in the outer portion of the globe, it can be drawn out with the point of a knife which has been "magnetized." This, of course, is not true, the fact being that when small particles are thus lodged in the cornea they are very firmly imbedded. They are usually so minute as to offer little or no attraction to the magnet and consequently they can be removed more promptly and satisfactorily with a needle point, than in any other way. But when a fragment of iron has been driven through the outer layers into the globe, then it becomes a serious question as to what methods can be employed for finding it, or drawing it out from the globe after it has been found. For this purpose the magnet is of undoubted value. Even small magnets of low power had served a good purpose in a limited way, a number of these having been proposed by different operators, each a modification of some previous form. The batteries used were of various patterns, but the electro-motive was small.

THE HAAB MAGNET.

The practical use of the magnet was restricted to a small class of cases, until at the suggestion of Prof. Haab, of Zurich, a decided step was made in advance. Although he employed the same principles in about the same way, the gain was by the larger scale adopted. His first magnet was described in the *Beitrage fur Augenheilkunde*, but the one I refer to has one or two further improvements on his model.

The core of this magnet is composed of soft iron of about twenty-four and one-fourth inches long, each end tapering to a point. Around this central core there is wound 5,387 feet of soft wire, arranged in the form of two spools. The core with the wire weighs 226 pounds. This is mounted on a heavy stand of oak, about five feet high. Unlike the original model also it is arranged with a swivel movement, permitting an adjustment of the point at any position. The current is supplied by the power line of the General Electric Company, which furnishes 500 volts, though it is necessary to reduce this to a pressure of 60 volts and a volume of eight ampères.

The very great power of this instrument can be estimated by a few simple experiments. If one holds out a small bunch of keys a short distance away, they apparently become animate and stand up, to reach out toward the magnet. If a watch be brought within a few feet of the machine, it at once becomes magnetised and must be passed through a tedious process of demagnetisation before it will keep time again. The force of the magnet is more exactly shown by the experiments made by Haab. He suspended a piece of iron, weighing one gram in front of the magnet by means of a thread. Another thread was attached to this gram of iron, this second thread running a short distance horizontally turned over a roller, and was attached below to a small disc, on which weights could be placed. It was thus possible to measure exactly the attractive force of the magnet, upon the gram of iron, and he found that when this gram of iron was at a distance of five millimeters from the magnet,

With a current of 6.6 amp. there was a drawing power of 133 grams.
With a current of 7.2 amp. there was a drawing power of 163 grams.
With a current of 7.9 amp. there was a drawing power of 213 grams.

Comparing this attractive power with any of the older and smaller magnets, they are like flint lock muskets compared with the modern thirteen inch rifles. So much for the magnet and its strength.

A word also as to the method of using it. The patient is seated in front of the instrument, and after the eye is under the influence of cocaine, one of the tapering ends is turned so as to bring the point of the core as near as possible to the eye. If a point is required which has a more acute angle, one tip can be replaced by another of any desired form. A weak current is then turned on and gradually increased in strength. Now, when a piece of iron has been lodged in the eye and no inflammation has as yet developed, the particle is drawn from the deeper portion, outward toward the magnet, and as it does so, striking the outer coating of the globe, the patient usually experiences a sharp pain at that point. This pain is of importance as showing the presence and location of the iron, and when thus moved it is often brought into a position where it can be seen more readily than before. If the point of entrance has not had time to close, that can be found, and the foreign body brought out through the same path by which it entered. In other cases it may be necessary to make a new opening, either near the point of entrance or opposite to that. In other words, each case is, to a certain extent a law unto itself. One point, however, is all important, which is that the case should be treated *as soon as possible* after the accident. Each hour at first is precious. Within a very short time, inflammatory changes begin, and the resulting exudation, with bands of adhesion, increase the difficulties in the removal. On one occasion, when a smaller magnet had failed to draw out a particle of iron which had been in the eye for some months I found that removal of the globe was necessary. After the eye was in a saucer, it was cut open, the particle was caught with the forceps, and so firm were the adhesions that as the iron was drawn away, nearly half of the interior of the globe came with it.

Although the magnet here described has been completed only about two weeks, an opportunity has already been afforded for testing its value in a case where it assisted very greatly in the removal of a piece of iron from the interior of the globe. As such accidents are constantly occurring in a manufacturing city like Buffalo, it seems proper thus to acquaint the profession here with the opportunity for saving eyes which would otherwise be lost, thus giving more prompt relief to our patients, and causing in some cases at least an ultimate saving to the treasury of the county.

183 Delaware Avenue.

## Figures and Tables

**Figure f1:**